# Applying the Vertically Integrated Project Model to Youth-Inclusive Social Media Coding of Zyn-Related YouTube Shorts: Methodological Case Study

**DOI:** 10.2196/95363

**Published:** 2026-09-02

**Authors:** Julia (Pengyue) Dou, Beth L Hoffman, Piper Narendorf, Tatiana Grinberg Limoncic, Grace Carver, Natalia Connor, Christine Larkin, Jaime E Sidani

**Affiliations:** 1 Department of Behavioral and Community Health Sciences School of Public Health University of Pittsburgh Pittsburgh, PA United States; 2 MYTH (Misinformation-identifying Youth in Tobacco and Health) Youth Collaborative Pittsburgh, PA United States

**Keywords:** collaborative coding, youth-inclusive research, social media content analysis, interrater reliability, vertically integrated project, VIP, nicotine and tobacco products

## Abstract

**Background:**

The rapid evolution of the nicotine and tobacco product (NTP) marketplace continues to outpace existing research. Although adolescents are active participants on social media who both consume and generate content, this age group has rarely been included in analyses of NTP content, raising a methodological challenge for accurate interpretation and coding of youth-oriented NTP content. Most research examining oral nicotine product content online has focused on TikTok, despite YouTube’s widespread adolescent use. One promising framework is the vertically integrated project (VIP) model. Situating content analysis within the VIP model suggests the methodological potential of age-diverse teams to capture nuances in social media data coding, reveal areas of interpretive divergence, and ultimately advance research methodologies in public health.

**Objective:**

This study aimed to understand how perspectives across different age groups, academic levels, and lived experiences shape the interpretation of Zyn-related YouTube shorts by engaging high school (HS) students, undergraduate public health students, and faculty researchers in parallel coding.

**Methods:**

We manually collected 300 publicly available YouTube shorts containing #Zyn on September 18, 2024, as a methodological case example. Three coder pairs, including 2 HS students (GC and NC), 2 undergraduate students (PN and TGL), and 2 public health faculty members (BLH and JES), independently coded the same 50 videos per round across 6 iterative rounds (N=300). For each round, interrater reliability was assessed using Cohen κ and percent agreement as complementary indicators. After all coding rounds were completed, the full coding team participated in a debriefing session. The transcript was analyzed using a combined deductive-inductive thematic approach to contextualize reliability patterns and identify challenges and practical insights related to age-diverse collaborative coding within the VIP-informed model.

**Results:**

Interrater reliability varied across 6 iterative rounds, with the undergraduate team showing the highest κ values in the final coding round (κ range: 0.40-0.63). Several constructs showed consistently high percent agreement but low and variable κ values, particularly within the HS team. For individual codes, mean κ values for male stereotype were higher for the undergraduate and faculty teams than for the HS team (0.56, 0.47, and 0.10, respectively). Debriefing findings indicated that disagreement reflected factors such as evolving code definitions, interpretive drift across rounds, and differences in cultural and experiential lenses. Coders noted that the codebook struggled to capture ambiguity in short-form content and that teams developed distinct heuristics for resolving uncertainty. Suggested improvements included rotating coding pairs and expanding codebook examples.

**Conclusions:**

This methodological case study illustrates how a VIP-informed, age-diverse coding structure can be used to examine interpretive challenges in coding youth-oriented nicotine-related short-form video content. For public health studies examining how different groups interpret online health messages, the VIP model may offer a practical structure for incorporating those perspectives into the research process.

## Introduction

### Social Media and Youth Nicotine and Tobacco Product Use

Prior research examining nicotine and tobacco product (NTP)–related social media exposure and e-cigarette use suggests that such exposure may shape youth perceptions and behaviors. A 2025 scoping review of 30 studies reported that most identified significant positive associations between NTP content exposure and e-cigarette use across diverse demographic groups [1].

The rapid evolution of the NTP marketplace continues to outpace existing research. While combustible cigarette use has declined, youth now encounter a broad array of alternatives, including oral nicotine products (ONPs), such as Zyn [2]. Unit sales of ONPs increased nearly 300-fold between 2016 and 2020 [3]. These tobacco-free, discreet pouches are available in youth-appealing flavors and are often marketed as “cleaner” alternatives [4]. Against this backdrop of increasing digital exposure and regulatory shifts following restrictions on flavored e-cigarettes [5], ONP manufacturers expanded their presence on digital platforms [6-8]. Such shifts raise concerns that nicotine use may be reintroduced to nicotine-naive adolescents through less overt, more culturally embedded pathways.

Despite a 2025 Pew Research Center survey indicating that more than 90% of teens aged 13-17 years use YouTube compared to 68% who say they use TikTok, to date, most research examining ONP content online has focused on TikTok [6,9,10]. The research has found that ONP manufacturers expanded their presence on digital platforms, often relying on youth-oriented cues such as lifestyle framing, humor, social appeal, and the minimization of health risks [6-10]. This is a notable gap in the literature, as about 96% of participants in a 2023 survey who reported current NTP use used YouTube, and 40% had seen YouTube content promoting NTP use [11]. Additionally, a previous qualitative study also found that YouTube videos were a main source of information about modifying e-cigarette liquids and devices [12]. Therefore, it would be valuable for researchers to extend the current literature examining ONP content on TikTok to YouTube.

Moreover, despite adolescents being active participants on social media who both consume and generate large volumes of content [13], to the best of our knowledge, this age group has yet to be included in analyses of NTP content on social media. Previous research has indicated that adolescents and adult researchers interpret health content on television differently [14], and a 2022 study that engaged young adults in the analysis of TikTok videos related to nicotine addiction reported differences in content analysis between young adults and the academic adult researchers [9]. These findings point to a potential methodological challenge regarding accurately interpreting and coding youth-oriented NTP social media content and suggest that when coding decisions rely solely on adult researchers’ perspectives or automated approaches, there is a substantial risk of misinterpreting the nuanced meaning of the content. Specifically, although the authors of the above-mentioned studies analyzing ONP content on TikTok noted that humorous and youth-appealing portrayals of Zyn were prevalent and could contribute to harm-minimization and normalization of Zyn use, it is unknown if adolescents and young adults, the primary audience for this content, would also classify this content as humorous and appealing to youth [6,9,10].

Considering these previous studies and the growing attention to NTP-related content on social media, it is prudent to begin to address this research gap and examine if there are differences between adolescent, young adult, and adult interpretations of this content [15].

### Vertically Integrated Project Model

One promising framework for examining this is the vertically integrated project (VIP) model. Originally developed at the Georgia Institute of Technology [16], the VIP model is a well-established approach that emphasizes long-term, faculty-led projects where students collaborate in multidisciplinary teams over multiple semesters or years [17,18]. Traditionally, VIP teams are led by 1 or more faculty members to facilitate greater mentorship and cross-collaboration. The VIP model attempts to create a supportable and long-lasting research community of faculty and students and can be used across various research modalities from basic science to community health [18]. This multigenerational approach facilitates knowledge transfer by enabling students to learn from and build upon the experience of more advanced scholars.

Regarding NTP content specifically, by incorporating youth as co-researchers within a structured, longitudinal framework, the VIP approach may enhance contextual interpretation of digital content while reducing interpretive bias. In other words, a VIP approach can allow for the examination of differences between adolescent, young adult, and adult interpretations of NTP social media content, as well as strengthen the interpretation of ambiguous NTP-related content, potentially identify engagement considerations among different audiences, and offer insight into how such material is targeted toward specific age segments.

Therefore, given this potential for a VIP approach to address the gap in adolescent participation in analyzing NTP content on social media and the gap in the literature regarding an examination of ONP content on YouTube, this study used a VIP model to conduct a qualitative analysis of ONP-related YouTube videos. By engaging high school (HS) students, undergraduate public health students, and faculty researchers in parallel coding, this methodological case study sought to understand how perspectives across different age groups, academic levels, and lived experiences shape the interpretation of Zyn-related YouTube shorts. Situating content analysis within the VIP model suggests the methodological potential of age-diverse teams to capture nuances in social media data coding, reveal areas of interpretive divergence, and ultimately advance research methodologies in public health.

## Methods

### Data Collection

Following discussions with the Misinformation-identifying Youth in Tobacco and Health (MYTH) Youth Collaborative, a youth participatory action research (YPAR) program that engages adolescents aged 13-17 years as partners in the examination of NTP-related social media content [19], we decided to narrow our analysis to YouTube shorts as MYTH members identified these as far more frequently viewed by their peers than longer form YouTube content.

To prepare HS coders who had no prior experience analyzing social media content and to retrain experienced coders, we first manually collected a time-stamped sample of YouTube shorts containing the hashtag #Zyn on September 5, 2024. This separate training set was used only for coder training and calibration and was not included in the analytic sample. Data collection was conducted on a single day, using the same device and geographic location, to capture a consistent platform snapshot. The search was conducted through the YouTube shorts search interface rather than the general YouTube search page. The researcher opened a new browser page without logging into a YouTube or Google account to reduce the influence of account-based personalization on the returned results, entered the search term “#Zyn,” and collected the first 100 publicly available shorts returned by the platform at the time of collection. No additional filters or sorting options were applied; videos were collected in the algorithmically ranked order returned by the YouTube shorts search interface. Because the videos were publicly available through the YouTube shorts interface, no private content was included. The research team then documented the video URLs for coding purposes, and videos were not downloaded or archived.

After completing the training and calibration process, we then collected a set of 300 YouTube shorts on September 18, 2024, for the main analysis, following the same search procedure described above.

Because this manuscript examines the VIP-informed coding process rather than estimating the prevalence of Zyn-related content on YouTube, the dataset serves as a methodological case example rather than a representative sample of all #Zyn content on YouTube shorts.

### Coding Team

To facilitate the VIP approach, this study was conducted by a coding team consisting of HS, undergraduate, and faculty coders. The team included 2 public health faculty members with over a decade of experience in social media and substance use research, and 2 undergraduate students with approximately 2-3 years of prior experience coding social media data related to NTPs and other health-related topics. Additionally, the team included 2 HS students (GC and NC) affiliated with the MYTH Youth Collaborative. One HS coder had experience in NTP-related coding before this study, while the other had no previous formal coding experience.

Furthermore, the team was characterized by high levels of educational attainment and institutional homogeneity: faculty and undergraduate coders were affiliated with the same academic institution and were trained in public health–related disciplines, and both HS coders attended well-funded local institutions. While this shared background supported efficient collaboration and a common research orientation, it also highlights the importance of reflexivity in considering how researchers’ social positions may shape the interpretation of youth-oriented social media content. The coding team was predominantly composed of individuals who identified as white women.

### Coding Procedures

For qualitative coding, we adapted an existing codebook previously used in research on e-cigarette-related social media [20,21]. The codebook was revised to reflect ONP/Zyn-specific terminology and short-form video cues, such as nicotine pouch packaging, visible pouch use, flavor references, comparisons with other nicotine products, Zyn-related humor, and youth or masculinity framing. The broader adapted codebook included multiple categories for coding ONP/Zyn-related short-form video content. Because the purpose of this methodological case study was to examine coding challenges rather than to report the substantive distribution of all coded content categories, the present analysis focused on a subset of constructs and individual codes that had been especially difficult for the larger research team to apply consistently in prior work and that were examined in both the interrater reliability (IRR) assessment and team debriefing.

These focal constructs included sentiment (pro-ONP, anti-ONP, and unclear), account type (the type of the account presenting the content), content type (the primary purpose and presentation style of the video), and appeal to groups of interest (stylistic, cultural, or narrative cues intended to resonate with particular audiences, including youth-oriented or gender-coded elements). These constructs were closely examined in both the quantitative assessment and the team debriefing session. Specific individual codes included youth appeal (features likely to appeal to youth, such as animation, humor, or popular cultural references), male stereotype (content invoking masculine characteristics or stereotypes, including male-coded interests or imagery), and personal experience (references to the poster’s own experience with Zyn/ONPs, including direct or implied personal use, but excluding proximal experience). Other coding categories included in the broader codebook were not specifically analyzed in the present study. An abbreviated supplemental codebook table is provided in Multimedia Appendix 1 for the focal constructs and key individual codes analyzed in this manuscript, including operational definitions, coding structures, and descriptive examples. Because the coded units were YouTube shorts, examples are provided as descriptive summaries of visual, audio, and narrative cues rather than original video clips, screenshots, usernames, or URLs.

The lead researcher for this study, PYD, oversaw all coding trainings, assignments, and adjudications. All 3 teams first participated in a structured training process. Each team independently coded a set of 100 #Zyn YouTube shorts using the adapted codebook. This practice round was not included in the analytic dataset but served as a calibration exercise to familiarize coders with the constructs, identify initial challenges, and establish baseline consistency. The main coding process was iterative and occurred across 6 rounds (N=300). In each round, 2 coders within each age group (HS, undergraduate, and faculty) independently coded the same set of 50 videos (“double-coded”). Following independent coding, all coders participated in a team debriefing session to reflect on coding discrepancies, interpretive challenges, and lessons learned throughout the coding process.

### IRR Assessment

To assess the consistency with which the 2 coders within each team applied the focal constructs and key individual codes to the 300-video analytic sample, IRR was assessed in Stata 19 (StataCorp LLC) using Cohen κ and percent agreement. For multicategory constructs (eg, sentiment: pro-ONP, anti-ONP, and unclear), mean κ for each construct and coding team was calculated by averaging all available category-specific κ values across categories and coding rounds. Given the relatively small size (N=300) of our dataset and the known limitations of κ in low-prevalence coding categories, percent agreement was used as a supplemental measure to provide a more nuanced view of consensus [22]. Commonly cited benchmarks (κ=0.70 and ≥80% agreement) were used as reference points to identify constructs that warranted closer review during adjudication, rather than as strict thresholds of acceptability [23]. IRR was calculated within coder groups (eg, HS-HS, undergraduate-undergraduate, and faculty-faculty). Reliability patterns were then compared descriptively across teams (HS, undergraduate, and faculty) to examine codebook consistency and identify areas that required further reflection during the team debriefing. Because each team included only 2 coders, these comparisons were not interpreted as evidence of group-level differences attributable to age, academic level, or lived experience.

### Team Debriefing and Reflective Analysis

After all 6 coding rounds were completed, we held a structured team debriefing session with all coders to discuss coding challenges, disagreements, and lessons learned from the VIP-informed coding process. We treated this session as a team reflection rather than a standalone qualitative study. The goal was to help us better understand the descriptive IRR patterns and identify practical changes that could improve future youth-inclusive social media coding projects.

The debriefing lasted approximately 90 minutes, was conducted via Zoom, and was facilitated by the lead author. All coders participated as members of the research team. A semistructured guide was used to organize the discussion, including reflections on teamwork across coding rounds, coding categories that were easier or more difficult to apply, constructs with lower agreement, and strategies for managing disagreements during adjudication.

The recording was then transcribed verbatim, after which the transcript was reviewed in Dedoose 10.34 (SocioCultural Research Consultants, LLC) using a deductive-inductive thematic approach [24]. Deductive coding was guided by a priori analytic anchors, including codes that showed consistently lower or more variable IRR in the quantitative analysis and recurring points of disagreement identified during adjudications across coding rounds. These anchors provided a structured way to examine coders’ reflections on interpretive challenges and connect them to patterns observed in the reliability results.

In parallel, inductive coding was used to capture themes that extended beyond these predefined areas, including reflections on age-based perspectives, learning across rounds, codebook limitations, interpretive drift, and collaborative dynamics across research teams. The lead author conducted the initial review and organization of the transcript. Because the debriefing was designed as a reflective team discussion rather than a standalone qualitative study, the transcript was not independently coded by multiple analysts, and formal intercoder agreement was not calculated. Themes were refined to capture both shared and divergent perspectives across the 3 age groups. All coder co-authors reviewed and confirmed that the final thematic interpretation accurately reflected the team debriefing. This combined deductive-inductive approach allowed the debriefing findings to contextualize the quantitative reliability patterns while identifying practical lessons for future age-diverse collaborative coding projects.

### Ethical Considerations

This study was conducted as part of a larger research project examining NTP messaging on social media. The overall research program was reviewed and approved by the Institutional Review Board (IRB) (Protocol #22080079). MYTH Youth Collaborative members and their parents or guardians provided assent and consent prior to joining the collaborative as part of our IRB protocol. Before the debriefing session, all coders were informed of its purpose and provided verbal agreement to participate and to be audio-recorded. As co-authors of this manuscript, the 2 HS coders (GC and NC) also reviewed and confirmed the thematic interpretation of the debriefing data, providing participant validation. Additionally, HS coders viewed the videos through their own YouTube accounts, so their access was limited by YouTube’s existing age-based restrictions. Videos requiring adult access or age verification would therefore not have been available to them during coding. We recognize that publicly available nicotine-related Shorts may still contain profanity, sexual innuendo, or normalization of substance use, and future youth-inclusive projects should build in clearer guidance for handling content that HS coders find uncomfortable.

For this study, we did not ask coders to disclose personal NTP use or personal exposure to NTP-related content, in order to avoid unnecessary disclosure of potentially sensitive information. Instead, we treated age, prior coding experience, and self-described cultural or experiential perspectives as reflexive factors that could shape interpretation and examined these factors through adjudication notes and the team debriefing.

In consultation with the IRB and consistent with the ethical criteria for internet research proposed by Eysenbach and Till [25], the present analysis involved publicly available social media content and did not involve human participants. Therefore, informed consent was not required. To protect anonymity, no usernames, profile descriptions, or identifiable metadata are reported.

## Results

### Quantitative IRR Results

#### Overall IRR Trends Across Coding Teams

Across 6 iterative coding rounds (N=300), Cohen κ values varied across rounds and coding teams (Table 1). Across the 6 rounds, κ values ranged from 0.30 to 0.54 for the HS team, from 0.40 to 0.63 for the undergraduate team, and from 0.47 to 0.61 for the faculty team. These ranges are presented descriptively and are not interpreted as evidence of group-level differences in reliability.

**Table 1:** Interrater reliability across coding rounds by coding team (N=300).

Team	κ range
HS^a^	0.30-0.54
UG^b^	0.40-0.63
Faculty	0.47-0.61

^a^HS: high school.

^b^UG: undergraduate.

#### Within-Team IRR for Focal Constructs

Within-team reliability for focal constructs was assessed across the 6 coding rounds for each team using both Cohen κ and percentage agreement as complementary indicators of consistency (Table 2).

**Table 2:** Summary of within-team interrater reliability across coding rounds by focal construct and coding team.

Construct and team			Mean κ (SD)^a^		κ range^b^		Agreement range (%)
Sentiment							
	HS^c^	0.43 (0.26)		0.37-0.58		80.67-94.33	
	UG^d^	0.64 (0.27)		0.43-1		85-95.29	
	Faculty	0.53 (0.29)		0.20-0.74		78.33-95.67	
Account type							
	HS	0.28 (0.34)		0-0.74		79.33-98	
	UG	0.39 (0.40)		0-0.75		84.33-98	
	Faculty	0.38 (0.38)		0-0.76		83-98	
Content type							
	HS	0.31 (0.27)		0.21-0.64		75.26-87.97	
	UG	0.50 (0.38)		0.07-0.86		88.33-93.33	
	Faculty	0.50 (0.37)		0.14-0.76		82-94.67	
Appeal to groups of interest							
	HS	0.11 (0.29)		0-0.10		79.23-98	
	UG	0.29 (0.46)		0-0.75		81-98.50	
	Faculty	0.38 (0.44)		0-0.67		82.67-99	

^a^For multicategory constructs (eg, sentiment: pro-ONP, anti-ONP, and unclear), mean κ and SD for each construct and coding team were calculated using all available category-specific κ values across categories and coding rounds.

^b^The κ range represents the minimum and maximum of the round-level construct κ values (ie, the average κ across the codes comprising the construct within each coding round) across the six coding rounds. Undefined κ values were excluded from the calculation of mean κ, SD, and κ range.

^c^HS: high school.

^d^UG: undergraduate.

This analysis focused on the reliability of focal constructs to capture how coder agreement evolved over time. For sentiment, mean κ values varied across teams, with the undergraduate team showing the highest mean κ (0.64, SD 0.27), followed by the faculty team (mean 0.53, SD 0.29) and the HS team (mean 0.43, SD 0.26). κ values also varied across rounds, ranging from 0.43 to 1 for the undergraduate team, 0.20 to 0.74 for the faculty team, and 0.37 to 0.58 for the HS team. Percentage agreement remained relatively high across all teams, ranging from 85% to 95.29% for the undergraduate team, 78.33% to 95.67% for the faculty team, and 80.67% to 94.33% for the HS team.

For account type, mean κ values were modest across teams (HS: 0.28, SD 0.34; undergraduate: 0.39, SD 0.40; faculty: 0.38, SD 0.38), although κ ranges varied across rounds. The HS team showed κ values ranging from 0 to 0.74, the undergraduate team from 0 to 0.75, and the faculty team from 0 to 0.76. However, percentage agreement remained consistently high across all teams, ranging from 79.33% to 98% for the HS team, 84.33% to 98% for the undergraduate team, and 83% to 98% for the faculty team.

For content type, mean κ values were higher for the undergraduate and faculty teams (mean 0.50, SD 0.38 for undergraduate; mean 0.50, SD 0.37 for faculty) than for the HS team (mean 0.31, SD 0.27). κ values also varied across rounds, ranging from 0.21 to 0.64 for the HS team, 0.07 to 0.86 for the undergraduate team, and 0.14 to 0.76 for the faculty team. Percentage agreement remained relatively high across all teams, ranging from 75.26% to 87.97% for the HS team, 88.33% to 93.33% for the undergraduate team, and 82% to 94.67% for the faculty team.

For appeal to groups of interest, mean κ values were highest for the faculty team (mean 0.38, SD 0.44), followed by the undergraduate team (mean 0.29, SD 0.46) and the HS team (mean 0.11, SD 0.29). κ values also varied across rounds, ranging from 0 to 0.10 for the HS team, 0 to 0.75 for the undergraduate team, and 0 to 0.67 for the faculty team. Despite these lower κ values, percentage agreement remained high across all teams, ranging from 79.23% to 98% for the HS team, 81% to 98.50% for the undergraduate team, and 82.67% to 99% for the faculty team. In some instances, complete agreement among coders produced undefined κ values. These values were excluded from the calculation of mean κ and SD, as well as from the reported κ ranges.

#### IRR for Key Individual Codes

Analyses of key individual codes were conducted to examine overall IRR for each team across coding rounds (Table 3). For these codes, mean κ and mean percentage agreement across rounds were reported to summarize overall coding consistency.

**Table 3:** Mean within-team interrater reliability for key individual codes by coding team.

Code and team		Mean κ (SD)^a^	Mean % agreement^b^
Youth appeal			
	HS^c^	–0.01 (0.01)	97
	UG^d^	0.28 (0.13)	81
	Faculty	0.44 (0.13)	82.67
Male stereotype			
	HS	0.10 (0.06)	79.20
	UG	0.56 (0.14)	81.33
	Faculty	0.47 (0.22)	88
Personal experience			
	HS	0.51 (0.35)	90.33
	UG	0.30 (0.39)	90.67
	Faculty	0.62 (0.18)	85

^a^For each individual code and coding team, mean κ was calculated by averaging round-level values across the six coding rounds; undefined κ values were excluded from both calculations.

^b^For each individual code and coding team, mean percent agreement was calculated by averaging round-level values across the six coding rounds.

^c^HS: high school.

^d^UG: undergraduate.

For youth appeal, the HS team had a near-zero mean κ (–0.01, SD 0.01), despite very high mean percentage agreement of 97%, suggesting that low code prevalence likely affected κ estimates. For male stereotype, mean κ values were higher for the undergraduate and faculty teams than for the HS team (mean 0.56, SD 0.14; mean 0.47, SD 0.22; and mean 0.1, SD 0.06 respectively), and percent agreement also remained higher for the undergraduate and faculty teams than for the HS team (81.33%, 88%, and 79.20%, respectively). For personal experience, mean κ values ranged from 0.30 (SD 0.39) to 0.62 (SD 0.18) across teams, and percent agreement remained high across all 3 teams, at or above 85%.

### Qualitative Results From Team Debriefing Session

The thematic analysis of the debriefing session revealed several key themes related to the collaborative coding process, highlighting the benefits, challenges, and nuanced interpretive differences that emerged across coding teams (Table 4).

**Table 4:** Themes from team debriefing session on the collaborative coding process.

Theme	Description	Illustrative quote
Interpretive drift and peer influence	Coders’ interpretations of the codebook evolved over time. Rather than relying solely on the codebook, coders often tried to anticipate how their partner would code, leading to “flip-flopping” definitions across rounds and creating a coding logic based on interpersonal dynamics.	“I kept putting what I thought the other faculty would think, you know, because I was always coding with the other faculty...you start to think just like them. You don't think of the code book.” (Faculty) “I was really remembering the conversations that we were having maybe even more so than the code book at times, because I knew that I was trying to think like the other coder…” (UG^a^)
Codebook limitations and tacit knowledge	The static codebook struggled to accommodate the novelty and ambiguity of short-form video content. Participants found it difficult to translate intuitive feelings about content into concrete, replicable coding rules.	“So much of it (the content) just to us, felt male stereotype for many different reasons, but we couldn't really define it to put it in the code book...” (UG) “I would feel like, okay, we've discussed everything we needed to discuss; we have very clear definitions, and then the next round would bring like, whole different type of videos that I hadn't really considered.” (Faculty)
Perceived role of cultural and experiential lenses	Coders described their interpretations as being influenced by cultural background, prior research experience, and lived experience. This was especially evident in the appeal to groups of interest construct.	“I think in that sense, it's very hard to define what is male stereotype. And I think with the other undergraduate coder and I specifically, of course, we had worked together before in the lab, but I feel like in this codebook in particular, our cultural differences really impacted how we saw it.” (UG) “I always have trouble trying to judge targeting of a group that I don't belong to...it also feels sometimes icky to me to try to code things that doesn't just make it seem like a terrible stereotype...” (Faculty)
Subjectivity of content-specific codes	Constructs such as sentiment, content type, and appeal to groups of interest were consistently challenging for all teams due to their inherent subjectivity.	“I remember at one point we sort of redefined comedy as it is supposed to be entertaining. And I think that helped. But...at first, I didn't code it as comedy, because I was like, it's not funny. But then it was like, okay, but they're trying to be funny.” (Faculty) “I feel like I struggled with the other substance and Zyn, because a lot of the videos we saw were talking about nicotine free pouches...my head would want to be like, oh, it's a Zyn, but then it wasn't.” (HS^b^) “...Initially, I would say she looks 15 to me, that's what I think. Um, after going to her account, sometimes they put their ages, they're like, oh, 23...” (UG)
Divergent heuristics between coder groups	The 3 teams developed different approaches to resolving ambiguity. Experienced faculty and UG coders often referred to definitions from previous projects, while the HS team used “fresh eyes” and concrete strategies like detailed notes to reach consensus more quickly.	“We were able to lean on some of that (prior experience)...the high school students...just got it really quickly...I think we were just kind of having analysis paralysis, right? Whereas they were able to come with fresh eyes too.” (Faculty) “...to always make notes on the certain sections so that we would establish, like, this one is only if they show Zyn in the video...adding notes and just kind of agreeing on certain things...definitely helped.” (HS)
Divergent emotional responses to content	The teams exhibited divergent emotional responses to the repetitive and often “annoying” video content. While the faculty team explicitly reported experiencing cognitive fatigue and frustration, the UG team developed humor as a coping mechanism.	“...just some of these videos were so annoying and so hard to watch, and I feel like maybe I could have done a better job if I was not just being like, oh my gosh, I can't watch another guy lifting weights with weird music in the background...” (Faculty) “...we just kind of took it (the video) with humor, and we were just seeing everything as, like, hallucination of videos, because some of them were so insane.” (UG)
Methodological suggestions for future projects	Participants proposed strategies to improve future projects, including re-pairing coders and enhancing the codebook with more specific examples to aid clarity.	“I do think it'd be interesting in the future if we did...the switching up of pairs...switching it up by age.” (Faculty) “...something that would be helpful is adding something in the example category...if there was an example, we could just easily refer back to that.” (HS)

^a^UG: undergraduate.

^b^HS: high school.

#### Interpretive Drift and Peer Influence

The coding process was not a static application of the codebook. Participants described a phenomenon of “interpretive drift” where their interpretations evolved over time, influenced by anticipating their partner’s logic. This created a coding logic based on interpersonal dynamics rather than relying consistently on the written codebook definitions. As one faculty coder noted, “I kept putting what I thought the other faculty would think… you start to think just like them. You don’t think of the codebook”.

#### Codebook Limitations and Evolving Definitions

Coders consistently expressed that the formal codebook struggled to capture the novelty and ambiguity of short-form video content. Participants found it difficult to translate intuitive feelings about content into concrete, replicable coding rules. A faculty coder explained, “I would feel like, okay, we’ve discussed everything... and then the next round would bring like, whole different types of videos that I hadn’t really considered”.

#### Perceived Role of Cultural and Experiential Lenses

Coders described their interpretations as being influenced by cultural background, prior research experience, and lived experience, particularly when applying subjective codes such as male stereotype. One undergraduate coder reflected on this, mentioning, “I feel like in this codebook in particular, our cultural differences really impacted how we saw it”.

#### Subjectivity of Content-Specific Codes

Several constructs (eg, content type and appeal to groups of interest) were consistently challenging for all teams due to their inherent subjectivity. For instance, a faculty coder shared the struggle with coding comedic video: “at first, I didn’t code it as comedy, because I was like, it’s not funny. But then it was like, okay, but they’re trying to be funny”.

#### Divergent Heuristics Between Coder Groups

The 3 teams developed different approaches to resolving ambiguity. The more experienced coders often relied on prior research strategies. In contrast, the HS team, without the burden of previous project methods, created their own concrete methods to reach consensus. A HS coder explained their approach, saying they learned to “always make notes on the certain sections so that we would establish, like, this one is only if they show Zyn in the video... adding notes and just kind of agreeing on certain things... definitely helped”.

#### Divergent Emotional Responses to Content

The teams exhibited different emotional responses to the repetitive and often “annoying” video content. The faculty team reported experiencing cognitive fatigue and frustration, with one member mentioning they “can’t watch another guy lifting weights with weird music in the background”. Conversely, the undergraduate team developed humor as a coping mechanism, with one coder saying, “we just kind of took it (the video) with humor... because some of them were so insane.”

#### Methodological Suggestions for Future Projects

Participants proposed several strategies to improve future collaborative coding projects. These suggestions included re-pairing coders to reduce interpretive drift and enhancing the codebook with more specific examples to aid clarity. One faculty coder suggested, “I do think it’d be interesting in the future if we did... the switching up of pairs... switching it up by age.”

## Discussion

This methodological case study examined how perspectives across different age groups, academic levels, and lived experience shaped the qualitative coding of youth-oriented short-form nicotine-related content within a VIP-informed, age-diverse coding structure. Rather than evaluating the prevalence or distribution of Zyn-related content, the study focused on the coding process itself, including descriptive IRR patterns and team reflections on interpretive challenges. Together, these findings illustrate how coder experience, age-related perspective, prior research exposure, and cultural lenses may shape the application of codes to ambiguous social media content.

The descriptive IRR patterns suggest that differences across teams may have reflected not only coding consistency, but also coders’ prior experience and familiarity with the content. The more experienced undergraduate and faculty teams showed higher κ values on complex, subjective constructs (eg, sentiment and content type). Their familiarity with established heuristics may have contributed to more consistent application of some codes. In contrast, the HS team demonstrated a notable learning curve, with IRR showing steady improvement across rounds. Their “fresh eyes” and development of concrete coding strategies appeared to help them apply the codes more consistently across rounds, particularly on youth-oriented content from a platform with which they were familiar.

The team debriefing findings helped contextualize the descriptive IRR patterns by suggesting that numerical disagreements reflected not only reliability challenges, but also the different forms of experience and perspective that coders brought to the collaborative structure of the VIP model [17]. For youth appeal, the near-zero κ among HS coders was likely influenced by the low prevalence of the code, as described in the quantitative results. The debriefing findings should therefore be understood not as an alternative explanation for the κ value, but as context for how coders understood and applied the youth appeal construct. Faculty coders expressed discomfort applying the code to a group to which they did not belong, whereas the HS team’s perspective as peers of the target audience likely shaped their application of the code differently. This suggests that the quantitative patterns were not simply measures of “correctness,” but rather reflected the distinct interpretive frameworks each team brought to the coding process. Consistent with the broader pattern observed for complex constructs, the higher κ values for male stereotype among the undergraduate and faculty teams may similarly reflect more stable within-pair coding heuristics and greater prior coding experience, rather than clearer or more accurate interpretation of the construct. The debriefing theme of Perceived Role of Cultural and Experiential Lenses helped explain why this code remained difficult, as coders from different cultural backgrounds interpreted masculinity cues differently. Fluctuations in IRR across rounds were explained by the themes of interpretive drift and codebook limitations. The team debriefing revealed that teams often developed their own internal logic and that the static codebook struggled to capture the nuances of fast-paced video content, leading to inconsistent codebook application. Finally, although personal experience showed moderate reliability differences across teams, this code was not a prominent topic during the debriefing session. Coders rarely reflected on challenges related to identifying personal experience narratives. This absence may partly reflect the relatively clear operational definition provided in the codebook, which specified explicit self-referential statements, making the code easier to apply consistently and less likely to become a major topic of discussion. Together, by integrating the descriptive IRR patterns with the team debriefing findings, these code-level examples illustrate a potential methodological benefit of age-diverse coding teams: they may help researchers recognize when constructs such as humor, youth appeal, masculinity, or harm-minimizing framing depend on audience-specific and culturally situated interpretations rather than on unambiguous content features. In future substantive content analyses, these interpretive differences could inform the refinement of codebook categories used to examine audience-oriented engagement cues; however, we also wanted to note that the present case study was not designed to infer creators’ intent, platform targeting, exposure effects, or associations with youth nicotine use.

This study has several limitations. First, the sample size of 300 YouTube shorts, while sufficient for a methodological exploration, limits the extent to which these coding-process insights can be generalized beyond this specific project. Our analysis was also confined to a single platform (YouTube) and a single hashtag (#Zyn); future research could benefit from including other short-form video platforms such as TikTok to capture a broader range of Zyn-related social media content.

Second, although the VIP model intentionally incorporated coders across multiple age groups, the coding team was relatively homogeneous. Coders generally had high levels of educational attainment or academic exposure, shared public health–related interests, and were predominantly White women. This shared background may have shaped how the team interpreted youth-oriented social media content, particularly for culturally specific or identity-related constructs. Future studies would benefit from more demographically and educationally diverse coding teams to further examine how coder positionality shapes collaborative coding processes and reliability patterns.

Third, although HS coders viewed the videos through their own YouTube accounts, we did not have a separate protocol for handling videos that HS coders might find uncomfortable. Future youth-inclusive coding projects should include clearer guidance for this situation.

Fourth, the team debriefing was facilitated and analyzed by the lead author, who also coordinated the coding process. Because the debriefing included HS, undergraduate, and faculty coders in the same discussion, participants may have been influenced by social desirability, team hierarchy, or group consensus. For this reason, we interpreted the debriefing findings as reflective team insights that help contextualize the coding process, rather than as generalizable evidence about how youth interpret Zyn-related content.

Finally, IRR was calculated using Cohen κ within fixed 2-coder teams and was used descriptively in this case study. Given the small coding team, the small number of videos per round, and the low prevalence of several codes, differences across teams should be interpreted only as case-based insights into how this specific team approached difficult-to-code Zyn-related YouTube shorts. Future reliability-focused studies, particularly those involving low-prevalence codes, may benefit from including Krippendorff α as an additional metric [26].

To our knowledge, this study is among the first to apply a VIP model to qualitative social media coding in public health. Our findings suggest that this approach is feasible and may be useful for identifying parts of the coding process that require additional clarification. In this study, when interpreted alongside the debriefing findings, lower IRR did not simply indicate poor reliability. We found that it also helped reveal where codebook definitions, coder assumptions, and team decision-making needed further discussion. However, persistent disagreement after training and adjudication should still be treated as a potential threat to classification validity and as a signal that code definitions, examples, or coder training require further refinement before substantive findings are interpreted. Overall, this perspective is especially important in studies of youth-oriented digital media, where meaning often depends on rapidly shifting humor, aesthetics, and platform-specific norms.

In addition to its methodological value, this study also highlights the educational and equity-related potential of adapting the VIP model for public health research. By extending a model originally designed for undergraduate training to include HS students as full analytic partners, the project created opportunities for early research skill development, mentorship, and exposure to scientific career pathways. At the same time, the team benefited from bidirectional learning in which the 2 HS coders (GC and NC) brought their own experiences as youth research partners, while the more experienced coders helped guide the coding process and codebook application. Future research could examine whether alternative team structures, such as cross-age coding pairs, help clarify how coders with different backgrounds resolve ambiguity during social media coding. Such designs may provide a more direct assessment of whether and how age-diverse collaboration shapes codebook refinement, adjudication, and consensus-building.

In conclusion, this methodological case study illustrates how a VIP-informed, age-diverse coding team can be used to examine interpretive challenges in coding youth-oriented nicotine-related short-form video content. The findings should be interpreted as case-based insights into one project’s coding process rather than as evidence that the VIP model improves reliability or generates more accurate interpretations. For public health studies examining how different groups interpret online health messages, the VIP model may offer a practical structure for incorporating those perspectives into the research process.

## Data Availability

The dataset analyzed in this study consists of publicly available YouTube shorts containing the hashtag #Zyn, manually collected on September 18, 2024. Due to platform dynamics and potential changes in content availability, direct redistribution of the original videos is not provided. The coding results that support the findings of this study are presented within the manuscript tables.

## Appendix

Multimedia Appendix 1 Abbreviated codebook for focal constructs and individual codes.

## Abbreviations

HS: high school
IRB: institutional review board
IRR: interrater reliability
NTP: nicotine and tobacco product
MYTH: Misinformation-identifying Youth in Tobacco and Health
ONP: oral nicotine product
VIP: vertically integrated project
YPAR: youth participatory action research
