# Supplementary material for: Applying the Vertically Integrated Project Model to Youth-Inclusive Social Media Coding of Zyn-Related YouTube Shorts: Methodological Case Study
Source: JMIR Infodemiology. 2026 Sep 2;6:e95363. doi: 10.2196/95363 (PMC13583466; doi:10.2196/95363)
Supplement: Supplementary file 1 [file infodemiology_v6i1e95363_app1.pdf]

Abbreviated Codebook for Focal Constructs and Individual Codes

This abbreviated codebook presents the constructs and individual codes from the full adapted codebook (originally developed for e-cigarette-related social media content and revised for ONP/Zyn-specific terminology and short-form video cues) that were the focus of the present methodological case study. Other categories included in the broader codebook were not specifically analyzed here and are not reproduced in this appendix. Each code was applied as a binary indicator (1 = present, 0 = absent) at the video level. Because the coded units were YouTube Shorts, examples below are provided as descriptive summaries of visual, audio, and narrative cues rather than as original video clips, screenshots, usernames, or URLs.

Table S1. Sentiment

Construct: the overall stance toward Zyn/ONPs expressed in the video.

**Code**	**Operational Definition**	**Coding Structure**	**Descriptive Example**
Pro-ONP	The video expressed a favorable stance toward Zyn or other ONPs. When multiple people expressed different views, the majority or dominant stance determined the code.	Mutually exclusive categorical construct. Coders selected one overall sentiment for each video; the selected category was coded 1 and the other categories 0.	A creator shown enjoying or casually incorporating the product into an activity in a positive, non-critical way.
Anti-ONP	The video expressed an unfavorable stance toward Zyn or other ONPs, including criticism, warnings, or encouragement to stop using them.	Same coding structure as above.	A video in which the creator explicitly discourages use of the product or highlights negative consequences of use.
Unclear	The video was insufficiently clear to classify as pro- or anti-ONP. This included content that lightly mocked Zyn without a strongly negative stance or presented both positive and negative views.	Same coding structure as above.	A creator describes both perceived benefits and drawbacks without endorsing or condemning the product.

Table S2. Account Type

Construct: the type of account presenting the content.

Code	Operational Definition	Coding Structure	Descriptive Example
Commercial/ Marketing	The video is authored by a supplier, distributor, retailer, or marketer that sold Zyn or other nicotine and tobacco products. Accounts selling only NTP-related accessories were not included.	More than one category could be selected when applicable; “Other” code included accounts that did not fit any of the named categories.	A nicotine retailer posts a Short displaying several pouch brands and directing viewers to purchase them.

Athlete	The video is authored by an individual identifiable as an athlete (professional, high school, or college level).	Same coding structure as above.	A poster whose profile or content identifies them as a competitive athlete in a traditional sport.
Appearance: adolescent	The post is authored by a person who appears to be a middle school or high school student or younger (i.e., under 18 years old). Coders may need to review the profile for additional information.	Same coding structure as above.	A poster whose appearance, school references, or profile details suggest they are a minor.
Appearance: young adult	The post is authored by a person who appears older than a middle/high school student but still appears to be a young adult (i.e., under 21 years old), such as a college student or recent graduate. Coders may need to review the profile for additional information.	Same coding structure as above.	A poster whose appearance and profile details (e.g., references to college) suggest a young-adult age range.
Other	The post is authored by any other type of account, including adult personal accounts, academic or government institutions, news accounts, and accounts that appeared to be automated or AI-generated.	"Other" code included accounts that did not fit any of the named categories.	A news organization posts a brief report about nicotine pouch sales.

Table S3. Content Type

Construct: the primary purpose and presentation style of the video (mutually exclusive categories).

Code	Operational Definition	Coding Structure	Descriptive Example
Informational	The primary purpose was to explain, educate, report, or provide commentary about Zyn/ONPs or another product. This included news or documentary-style content and personal narratives when the main purpose was to inform.	Mutually exclusive categorical construct. Coders selected one primary purpose for each video; the selected category was coded 1 and the other categories 0.	A video in which the creator explains how the product works or reviews it in an explanatory, non-comedic format.
Comedic	The primary purpose was to entertain through humor, a skit, a meme format, parody, or a humorous interview/podcast excerpt. The code reflected the apparent content intent to be funny.	Same coding structure as above.	A meme-style skit exaggerates how someone behaves after taking a pouch, using comedic audio and captions.

Product	The primary purpose was to display a product or related item, such as multiple cans, an unboxing, or a Zyn-branded accessory, with little or no explanatory commentary.	Same coding structure as above.	A video showing the product itself (including branded merchandise) with no additional commentary.
Other	The video's primary purpose did not fit the informational, comedic, or product-focused categories. This included tricks involving Zyn, requests for information, and other uses of the product or packaging.	Same coding structure as above.	A creator demonstrates a trick using a pouch can or asks viewers how to redeem reward points.

Table S4. Appeal to Groups of Interest

Construct: stylistic, cultural, or narrative cues intended to resonate with/appeal to particular audiences. The youth-appeal and male-stereotype codes within this construct were additionally examined individually (see note below).

Code	Operational Definition	Coding Structure	Descriptive Example
Youth Appeal*	Content with features likely to appeal to a youth audience, such as animation, colorful packaging or flavor cues, background music, humor, wordplay on the brand name, or popular cultural references (e.g., a well-known podcast, fraternity culture) targeted at youth or young adults.	More than one category could be selected when applicable; codes were based on what appeared in the video, rather than assumptions.	A brightly colored, animated video with upbeat background music and a pun on the brand name.
Black Representation/ Potential Appeal	A person shown in the video was perceived by the coder as Black, which the original codebook treated as a possible cue for appeal to Black audiences.	Same coding structure as above.	A video features a person perceived by the coder as Black discussing or using Zyn.
Hispanic Representation/ Potential Appeal	A person in the video appeared Hispanic/Latino or the video used cultural cues interpreted as appealing to Hispanic/Latino audiences, such as Latin music.	Same coding structure as above.	A creator perceived as Hispanic/Latino discusses Zyn while Latin music plays in the background.
Sexual or Gender Minority Appeal	The video included explicit LGBTQ+ references or cues, such as rainbow or Pride-themed imagery or products.	Same coding structure as above.	A Short shows Pride-themed packaging and includes rainbow imagery and an LGBTQ+ reference in the caption.
Male/ Masculine Stereotypes*	The video included cues that coders interpreted as appealing to male audiences or drawing on masculine stereotypes, such as trucks, construction or cowboy themes, gym	Same coding structure as above.	A group of men at a gym use pouches while the video emphasizes strength, toughness, and "gym bro" culture.

	or fraternity culture, and male-oriented podcasts.		
Female/ Feminine Stereotypes	The video included cues that coders interpreted as appealing to female audiences or drawing on feminine stereotypes, such as “get ready with me,” beauty, lifestyle, or day-in-the-life content framed for girls or women.	Same coding structure as above.	A video using a “get ready with me” or “day in the life” format typically associated with teenage girls or women.
Military or law-enforcement appeal	The video included cues that coders interpreted as appealing to military or law-enforcement audiences, such as military or police figures, uniforms, imagery, language, or hashtags.	Same coding structure as above.	A video using hashtags referencing the military.

**Youth and male/masculine stereotypes were the two codes from this construct examined individually for inter-rater reliability, alongside personal experience (below).*

Table S5. Personal Experience

Individual code examined in both the reliability assessment and team debriefing.

Code	Operational Definition	Coding Structure	Descriptive Example
Personal Experience	The video described or implied the poster’s own experience with Zyn or other ONPs. This included visible use, such as placing a pouch in their mouth, or other indications that the poster used the product. Experiences involving only someone else’s use were not included.	Binary code applied independently to the code (1 = present, 0 = absent).	The creator describes how Zyn feels for them and is shown placing a pouch in their mouth.

Note. ONP=oral nicotine product; NTP=nicotine and tobacco product.
